# A systematic review of heart rate variability and menopausal vasomotor symptoms

**DOI:** 10.14814/phy2.70907

**Published:** 2026-05-12

**Authors:** Rashmin Hira, Jaiden Uppal, Paras Deol, Danaka Porter, Derek Exner, Satish R. Raj, Jacquie R. Baker

**Affiliations:** ^1^ Libin Cardiovascular Institute University of Calgary Calgary Alberta Canada; ^2^ Department of Cardiac Sciences University of Calgary Calgary Alberta Canada

**Keywords:** autonomic nervous system, cardiovascular disease risk, heart rate variability, menopause, vasomotor symptoms

## Abstract

The menopausal transition is associated with increased cardiovascular disease (CVD) risk. Vasomotor symptoms (VMS) are highly prevalent during this period, potentially due to autonomic nervous system (ANS) changes. This systematic review examines whether heart rate variability (HRV), a noninvasive marker of autonomic function, differs between women with and without VMS. Five databases were searched. Studies were included if they assessed HRV in women with and without VMS while in peri‐ and/or post‐menopause. Means, standard deviations, and sample sizes were extracted. Pooled standardized mean differences were calculated using a random‐effects model with restricted maximum likelihood estimation. Heterogeneity was assessed using Cochrane's Q and *I* ^2^ statistics. Three studies were suitable for meta‐analysis. The pooled analysis revealed no difference in low frequency (LF) in women with VMS compared to those without VMS (Difference in means: 2.715 [95% CI: −0.124 to 5.555]; *p* = 0.06). There were no group differences in high frequency (HF) (*p* = 0.11). High heterogeneity was observed in both LF (Q = 73.96, *p* < 0.001, *I* ^2^ = 97%) and HF (Q = 61.82, *p* < 0.001, *I* ^2^ = 97%). Women with VMS and without VMS did not show differences in HRV. Larger, standardized studies are needed to better understand the relationship between autonomic function, VMS, and CVD risk.

## Graphical abstract

Systematic evaluation of differences in heart rate variability (HRV) between women with and without vasomotor symptoms (VMS) while in peri‐ or post‐menopause. Two out of the three eligible studies found differences in LF and LF/HF, but the pooled analysis showed no statistical significance between women with and without VMS (*p* = 0.06).

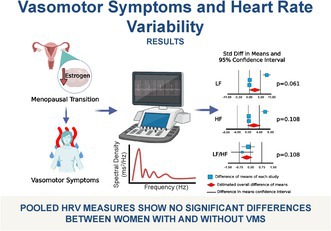

## 1 INTRODUCTION

Cardiovascular disease (CVD) is the leading cause of death for women globally (Tsao et al., 2023). Despite this, CVD risk in women is often underestimated, partly due to the longstanding misconception that women are uniformly protected from heart disease during their reproductive years (Maas & Appelman, 2010). While estrogen appears to exert cardioprotective effects in younger women, this benefit diminishes significantly with age and after menopause (Rosano et al., 2007). As estrogen levels fall during the menopausal and post‐menopausal periods (Motlani et al., 2023), heart disease risk rises substantially, with the incidence of heart failure comparable between post‐menopausal women and men (El Khoudary et al., 2020; Lam et al., 2019).

One of the most commonly reported symptoms during the menopause transition is the onset of vasomotor symptoms (VMS), including hot flashes and night sweats (Gold et al., 2000; Kronenberg, 1990). These symptoms are not only bothersome but have also been associated with reduced quality of life and potential negative health outcomes (Özkaya et al., 2011; Thurston et al., 2011). Emerging evidence suggests a possible association between VMS and increased CVD risk (Thurston et al., 2016). Despite this, direct comparisons between VMS and cardiovascular outcomes are sparse, highlighting the need to investigate the physiological mechanisms that may underlie this relationship.

The autonomic nervous system (ANS), which regulates involuntary functions including cardiovascular control and thermoregulation, represents a plausible physiological pathway linking VMS and cardiovascular risk (Benarroch, 2020). In menopausal women, several studies have suggested altered autonomic balance, with evidence of increased sympathetic activity and reduced parasympathetic activity (Thurston et al., 2012; Tuomikoski et al., 2011). Additionally, hot flushes themselves are associated with transient surges in sympathetic activity (Lee et al., 2022; Low et al., 2011), mediated through sympathetic cholinergic sudomotor pathways (Archer et al., 2011). Thus, VMS may reflect repeated episodes of heightened sympathetic drive. Whether this autonomic imbalance and the cumulative burden of sympathetic surges contribute causally to elevated CVD risk remains uncertain.

Measurements such as blood pressure, heart rate, and heart rate variability (HRV) are often used as proxies for ANS activity. HRV offers valuable insights into sympathetic and parasympathetic balance (Shaffer & Ginsberg, 2017). Both time and frequency domain parameters of HRV help delineate ANS function, providing a framework for assessing whether ANS regulation differs between women with and without VMS (Bigger et al., 1992; Hillebrand et al., 2013; La Rovere et al., 1998; Wolf et al., 1978). To explore whether autonomic dysfunction differs between women with and without VMS, we systematically evaluated differences in HRV between women with and without VMS while in peri‐ and/or post‐menopause.

## 2 METHODS

A systematic review was conducted following the guidelines outlined in the Preferred Reporting Items for Systematic Reviews and Meta‐Analyses (PRISMA) (Page et al., 2021) (Table S1). This study was exempt from Institutional Review Board approval. Data for this study are available from the authors upon reasonable request.

### 2.1 Search strategy

The following databases were systematically searched without language restriction from inception to March 2026: Excerpta Medica Database (Embase), Medical Literature Analysis and Retrieval System Online (MEDLINE), Scopus, Web of Science, and Cumulative Index to Nursing & Allied Health Literature (CINAHL). Databases were searched for studies reporting vasomotor symptoms (VMS) and heart rate variability (HRV) outcomes in adult women. The Medical Subject Headings terminology and keywords were related to “hot flashes”, “night sweats”, “heart rate variability”, and “menopause”.

### 2.2 Study selection

Titles and abstracts of potentially eligible articles were independently screened by three reviewers (DP, JU, and PD). Subsequently, three reviewers (DP, JU, and PD) separately reviewed the full text of potentially eligible articles. All randomized and non‐randomized controlled trials, cohort studies, correlational studies, and case studies (*n* ≥ 3) that enrolled midlife women (35–65 years) with and without VMS (daytime hot flashes/flushes and night sweats) were included. The “with VMS” group served as the treatment group and the “without VMS” group served as the control group. All selected studies must have established the presence or absence of VMS using either structured questionnaires or self‐reported symptoms and have collected measures of HRV for a minimum 5‐min period (range 5‐min–24 h recordings). Studies were excluded if they included patients with premature ovarian failure or hot flashes resulting from medical treatments (e.g., chemotherapy, fertility treatments, and Leuprolide). All inter‐reviewer conflicts were resolved by an additional reviewer (JB).

### 2.3 Quality assessment

Quality assessment for each study was performed by two independent reviewers (JU and PD) using the Newcastle‐Ottawa Grading Scale (NOS) (Wells et al., n.d.). Studies were assessed according to selection criteria (4 points), comparability (2 points), and outcome measures (3 points), totaling 9 points. Study quality assessment was rated as poor (0–3), fair (4–6), or good (7–9). All conflicts were resolved by a third reviewer (JB).

### 2.4 Data extraction and analysis

Data were independently extracted from selected studies by two reviewers (JU and PD) using a predesigned extraction tool. Extracted data included: study details (author, year, language, study design, menopausal stage [e.g., peri‐menopausal vs. post‐menopausal], description of comparator group, inclusion/exclusion criteria, and HRV collection details), and study participant characteristics (*n*, %, age, symptom severity [e.g., mild, moderate, and severe]). Primary outcomes included time domain (standard deviation of NN and RR intervals [SDNN], root mean square of successive differences [RMSSD]) and/or frequency domain (i.e., low frequency power [LF], high frequency power [HF], and LF/HF ratio) parameters of HRV. When data were presented only in figure form, primary outcomes were extracted using publicly available software (WebPlotDigitizer; https://automeris.io/WebPlotDigitizer). Discrepancies between extracted data were resolved by an additional reviewer (JB).

### 2.5 Statistical analysis

Means, standard deviations (SDs), and sample sizes for HRV parameters were extracted from each study (*n* = 3). For pooled analyses, relevant study means, SDs, and sample sizes for HRV parameters in patients with and without VMS were entered into Comprehensive Meta‐Analysis (CMA) Software Version 4 (Biostat, Englewood, NJ) to provide a standardized difference of means. Difference in means was then compared using a random‐effects model (restricted maximum likelihood estimation method, inverse variance weight) with 95% confidence intervals, and figures were generated using the CMA software (Biostat, Englewood, NJ). Study heterogeneity was estimated using Cochrane Q (*p* < 0.05) and I^2^ statistic. A Q test with a *p* < 0.05 suggests the degree of heterogeneity is beyond random chance or error (Higgins et al., 2003). *I* ^2^ < 25% was considered low heterogeneity, 25% to 50% moderate, and >50% high (Higgins et al., 2003).

## 3 RESULTS

### 3.1 Study selection and characteristics

**FIGURE 1:**
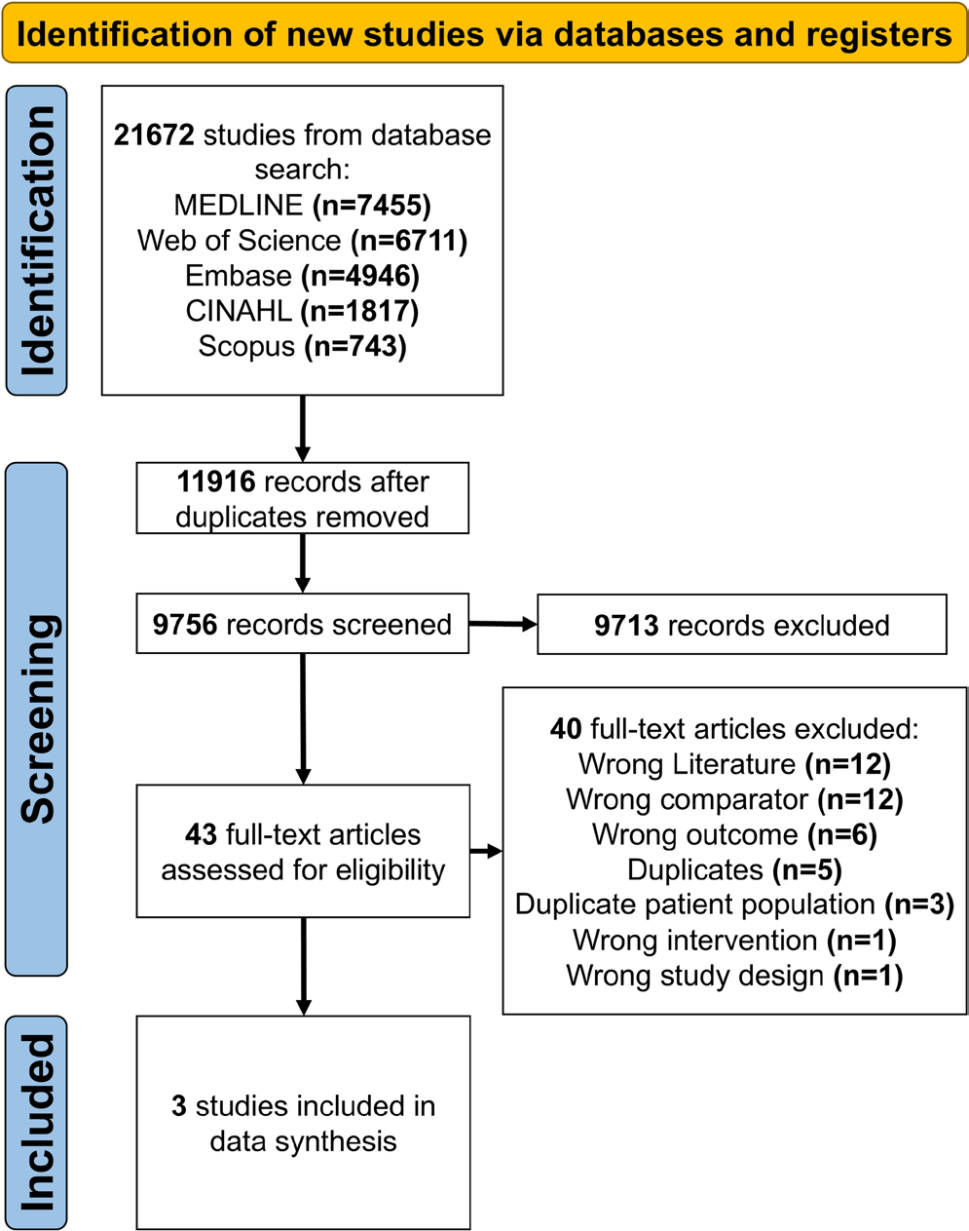
Preferred Reporting Items for Systematic Reviews and Meta‐Analyses (PRISMA) flow diagram for systematic review and meta‐analysis.

The study selection process is shown in Figure 1. A total of 21,672 studies were identified (MEDLINE *n* = 7455; Embase *n* = 4946; Web of Science *n* = 6711; Scopus *n* = 743; CINAHL *n* = 1817). Following removal of duplicate studies (*n* = 11,916), 9756 titles and abstracts were screened. A total of 9713 articles were excluded, with a 99% (9670/9756) reviewer agreement. The remaining 43 full‐text articles were reviewed, with three studies meeting the inclusion criteria for data synthesis; reviewer agreement was 77% (33/43). Across the three studies, a total of 231 study participants were reported, with a total of 187 participants reported to have VMS versus forty‐four participants without VMS. Sample sizes ranged from 36 to 150 participants. Study characteristics are summarized in Table 1. All three articles were deemed to be of good quality (Maas & Appelman, 2010; Rosano et al., 2007; Tsao et al., 2023) (Table 2).

**TABLE 1:** Included study characteristics.

Study	Study design	No VMS n	VMS n	Control cohort	Comparator	Location of study	Age range (years)	Body mass index (kg/m^2^)	Main result	Reporting method	Intervention duration
Akiyoshi et al. (2011)	Non‐Randomized Control	11	34	No VMS	Patient with VMS	Japan	50.9 ± 0.5	22.0 ± 0.3	Observed a decrease in HRV (LF and total power) in women with VMS.	Self‐reported VMS symptoms and structured questionnaires	5 min
Hoikkala et al. (2010)	Non‐Randomized Control	23	127	No VMS	Patient with VMS	Finland	48–55 No VMS: 53 ± 2 Mild VMS: 53 ± 2 Moderate VMS: 52 ± 2 Severe VMS: 52 ± 2	No VMS: 23 ± 3 Mild VMS: 23 ± 2 Moderate VMS: 23 ± 2 Severe VMS: 23 ± 2	Observed that RMSSD was significantly lower in women with VMS compared to without. LF and HF experienced a decreasing trend, although insignificant. This may indicate an increase in sympathetic activity and a decrease in parasympathetic activity.	Self‐reported VMS symptoms and Hot Flash Weekly Weighted Symptom Score	24 h
Lee et al. (2011)	Non‐Randomized Control	10	26	No VMS	Patients with VMS	Korea	No VMS: 50.20 ± 0.85 VMS: 51.42 ± 0.49	No VMS: 25.48 ± 4.17 VMS: 23.06 ± 2.02	Observed that the LF/HF ratio increased with severity of hot flashes, possibly suggesting greater sympathetic nervous system dominance with VMS. HF trended lower in women with moderate‐to‐severe hot flashes, but was insignificant.	Self‐reported VMS symptoms and Korean MRS	5 min

Abbreviations: HF, high frequency; HRV, heart rate variability; LF, low frequency; MRS, menopause rating scale; RMSSD, root mean square of successive differences; VMS, vasomotor symptoms.

**TABLE 2:** Quality assessment according to Newcastle‐Ottawa Scale.

	Akiyoshi et al. (2011)	Hoikkala et al. (2010)	Lee et al. (2011)
Selection (/4)			
Cohort representativeness	1	1	1
2 Selection of controls	1	1	1
3 Exposure ascertainment	1	1	1
4 Adequate baseline period	1	0	1
Selection total	4/4	3/4	4/4
Comparability (/2)			
Study excludes conditions that affect primary outcomes	1	1	1
2 Study excludes for medications affecting primary outcomes	1	1	1
Comparability total	2/2	2/2	2/2
Outcome measures (/3)			
Primary outcome assessment	1	1	1
2 Adequate observation period	1	1	1
3 Completeness	1	1	1
Outcome measures total	3/3	3/3	3/3
NOS TOTAL (/9)	9	8	9

### 3.2 Frequency domain analysis of heart rate variability in women with and without VMS

**FIGURE 2:**
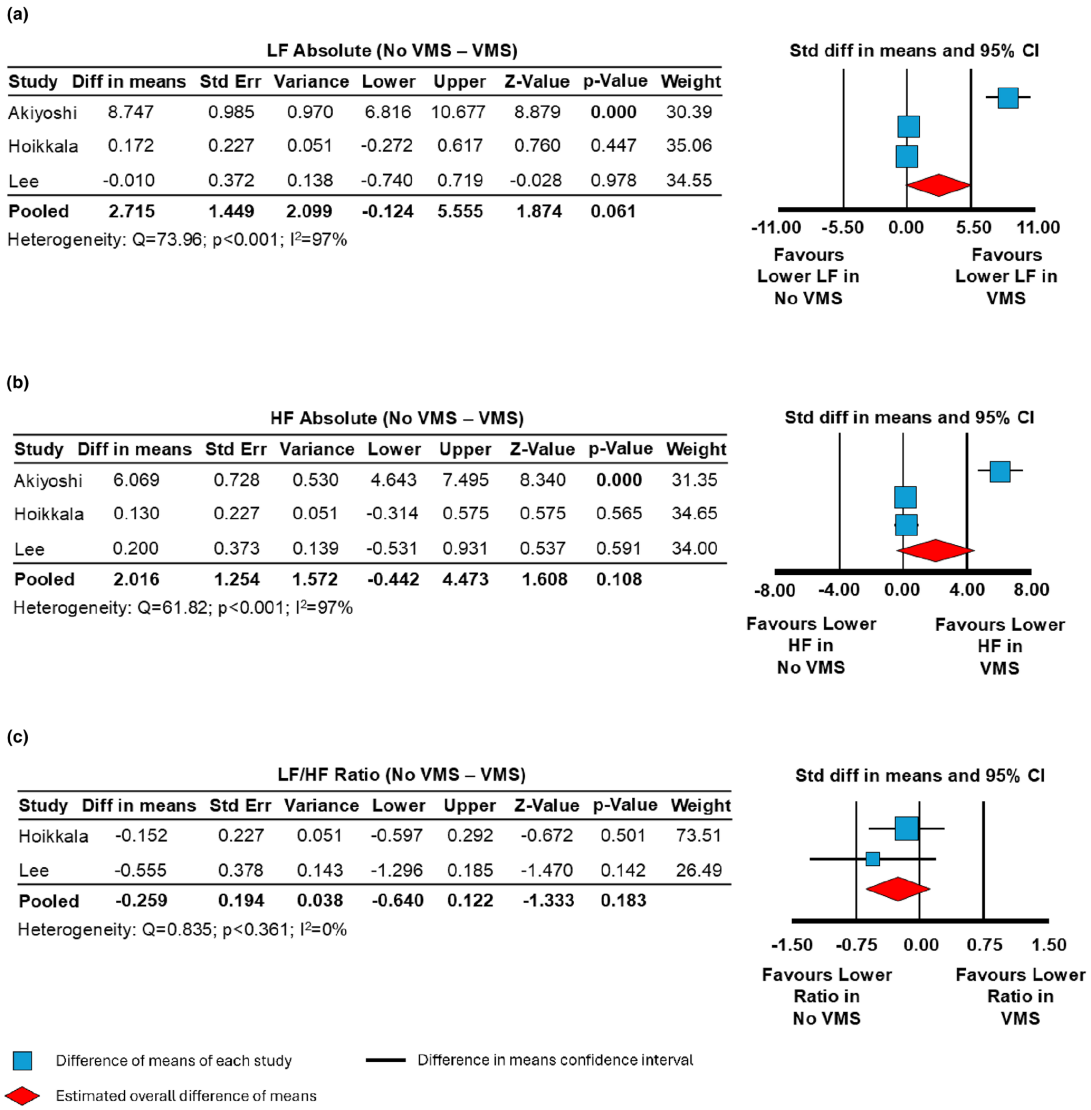
Forest plots of difference in means for heart rate variability frequency domain parameters in women with (*N* = 187) and without (*N* = 44) vasomotor symptoms (VMS). (a) Low frequency power (LF) in women with no VMS versus with VMS (*p* = 0.061). (b) High frequency power (HF) in women with no VMS versus with VMS (*p* = 0.108). (c) LF/HF ratio in women with no VMS versus with VMS (*p* = 0.183).

While one study (Akiyoshi et al., 2011) reported significantly lower absolute LF and HF power in women with VMS compared to those without (*p* < 0.001), the pooled analyses revealed no overall differences for either LF (*n* = 3, Difference in means: 2.715 [95% CI: −0.124 to 5.555]; *p* = 0.06; Figure 2a) or HF power (*n* = 3, Difference in means: 2.016 [95% CI: −0.442 to 4.473]; *p* = 0.11; Figure 2b). High heterogeneity was observed in both LF (*Q* = 73.96, *p* < 0.001, *I* ^2^ = 97%) and HF power (HF: *Q* = 61.82, *p* < 0.001, *I* ^2^ = 97%).

The absolute LF/HF ratio also showed no difference between groups (*n* = 3, Difference in means: −0.259 [95% CI: −0.640 to 0.122]; *p* = 0.18; Figure 2c), with low study heterogeneity (*Q* = 0.835, *p* = 0.361; *I* ^2^ = 0%). Normalized HRV parameters are presented in Figure S1. No significant differences were observed between women with and without VMS.

### 3.3 Time domain analysis of heart rate variability in women with and without VMS

**FIGURE 3:**
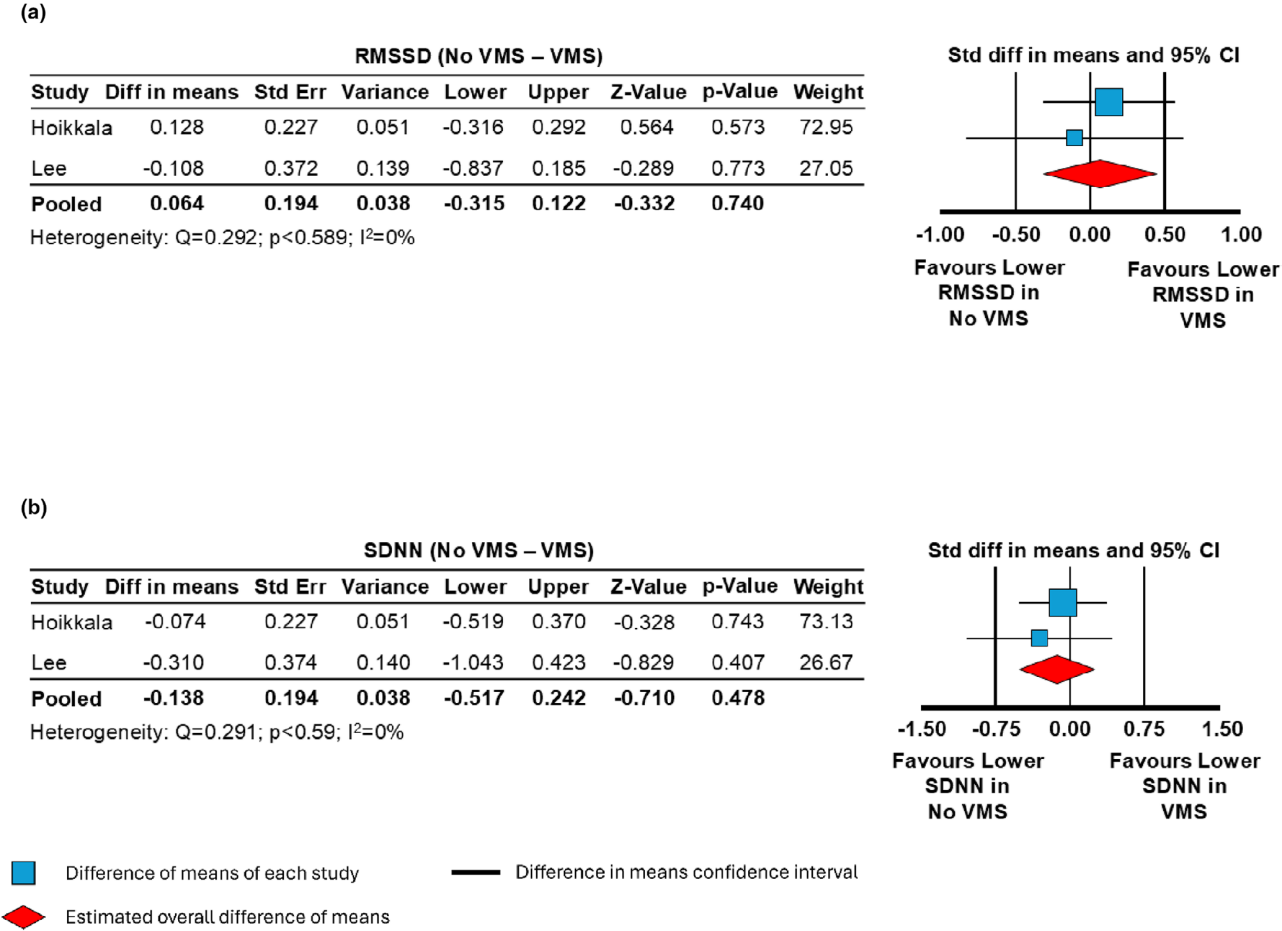
Forest plots of difference in means for heart rate variability time domain parameters in women with (*N* = 187) and without (*N* = 44) vasomotor symptoms (VMS). (a) Root mean square of successive differences (RMSSD) in women with no VMS vs. with VMS (*p* = 0.740). (b) Standard deviation of the NN (normal‐to‐normal) intervals (SDNN) in women with no VMS versus with VMS (*p* = 0.478).

Compared to women without VMS, individuals with VMS had no significant differences in either RMSSD (*n* = 3, Difference in means, −0.064 [95% CI, −0.315 to 0.444]; *p* = 0.74; Figure 3a) or SDNN (*n* = 3, Difference in means, −0.138 [95% CI, −0.517 to −0.242]; *p* = 0.48; Figure 3b). Low heterogeneity was detected in both RMSSD (*Q* = 0.292, *p* = 0.29; *I* ^2^ = 0%) and SDNN (*Q* = 0.835, *p* = 0.59; *I* ^2^ = 0%).

## 4 DISCUSSION

Increased CVD risk in menopausal and post‐menopausal women has prompted growing interest in identifying contributing physiological mechanisms. Vasomotor symptoms (VMS) are highly prevalent during the menopausal transition and may reflect underlying autonomic changes relevant to CVD risk. Here, we aimed to systematically review and analyze the potential relationship between VMS and autonomic impairments using HRV as a surrogate measure of autonomic control. Among the three studies that met the inclusion criteria, one reported heightened LF power (Akiyoshi et al., 2011) and one found higher LF/HF (Lee et al., 2011) in women with VMS (suggestive of autonomic imbalance), and one found no significant differences between groups (Hoikkala et al., 2010). Overall, our pooled analysis revealed no statistically significant difference (*p* = 0.06) in LF between women with and without VMS.

Akiyoshi et al. (2011) examined cardiac autonomic function in 45 Japanese peri‐ and postmenopausal women (Table 1). The researchers evaluated the relationship between HRV and VMS by comparing women with VMS (specifically hot flashes or sweating) to asymptomatic women (Akiyoshi et al., 2011) (Table 1). HRV was measured using power spectral analysis applied to 5‐min continuous thoracic electrocardiograms. The peri−/postmenopausal women with VMS had lower resting LF power and HF power, indicative of lower cardiac parasympathetic and sympathetic function compared to women without VMS (Akiyoshi et al., 2011). Further, menopausal women with VMS had higher mean heart rates and systolic and diastolic blood pressure compared to women without VMS (Akiyoshi et al., 2011). The researchers suggest the vascular sympathetic nervous system was playing a compensatory role in response to the reduced cardiac parasympathetic function (Akiyoshi et al., 2011).

Similarly, Lee et al. (2011) investigated the relationship between VMS and HRV in 36 postmenopausal women (Table 1). They also measured HRV using a 5‐min electrocardiogram recorded in the resting state. The researchers found the LF/HF ratio to be significantly higher in postmenopausal women with VMS compared to women without VMS (Lee et al., 2011). A higher LF/HF ratio was used as an indicator of balance between the sympathetic and parasympathetic nervous systems, with an increase in the ratio indicating increased sympathetic function (Shaffer & Ginsberg, 2017).

Conversely, Hoikkala et al. (2010) analyzed 24‐h ambulatory electrocardiographic recordings from 150 postmenopausal women with varying degrees of hot‐flash severity as well as women without any VMS (Table 1). While the researchers found no associations between frequency domain HRV markers (e.g., LF power and HF power) and the overall frequency and severity of VMS, they found that RMSSD was significantly lower in women with VMS (Hoikkala et al., 2010). Reduced RMSSD is indicative of reduced parasympathetic function (Shaffer & Ginsberg, 2017).

### 4.1 Clinical implications

Out of the three studies, one found lower LF, one found higher LF/HF, and one found lower RMSSD between women with and without VMS. Differences found in separate HRV parameters may have resulted in the insignificant pooled analysis.

In the frequency domain analysis, LF power (0.04–0.15 Hz) reflects oscillations in heart rate that are mediated by both sympathetic and parasympathetic branches of the autonomic nervous system (Malik et al., 1996), but its interpretation is complex and multifaceted. LF is also strongly influenced by baroreflex sensitivity, the homeostatic mechanism that adjusts heart rate in response to blood pressure changes (La Rovere et al., 1998). A reduction in LF power or LF/HF may reflect an imbalance between sympathetic and parasympathetic activity, as well as diminished baroreflex function, a marker of impaired cardiovascular adaptability (La Rovere et al., 1998). Lower LF power and RMSSD have been associated with increased cardiovascular risk, including adverse outcomes post‐myocardial infarction and higher mortality in heart failure cohorts (Kleiger et al., 1987; La Rovere et al., 1998). Although our pooled analyses did not illustrate statistically significant reductions in LF between women with and without VMS, the trend observed in the available data underscores the need for continued investigation into whether subtle autonomic shifts accompany VMS.

Only three studies met the inclusion criteria. If these results are reproduced in future larger cohorts of patients and with standardized data collection (i.e., over 5 min baseline periods (Shaffer & Ginsberg, 2017)), HRV may serve as an early, non‐invasive biomarker of autonomic health in women with VMS. This could inform risk stratification models that better account for sex‐specific autonomic pathways in cardiovascular aging. VMS are highly prevalent yet underrecognized in CVD screening. These results highlight a broader need to assess the relationship between estrogen decline and VMS.

### 4.2 Limitations

Several limitations must be considered when interpreting these findings. The primary limitation was the overall scarcity of existing literature. Despite the clinical relevance of VMS, autonomic health, and heightened CVD risk in menopausal women, only three studies met the inclusion criteria for pooled analysis. Additionally, several studies in this field relied on overlapping cohorts (Akiyoshi et al., 2011; Jones et al., 2016; Lee et al., 2011; Martinelli et al., 2020; Neufeld et al., 2015; Salin et al., 2022; Thurston et al., 2011, 2016) raising concerns surrounding redundancy and the generalizability of findings to broader menopausal populations. Moreover, these studies significantly lacked diversity in their cohort characteristics, including women only from Finland (Hoikkala et al., 2010), Japan (Akiyoshi et al., 2011), or Korea (Lee et al., 2011). This is especially problematic, as VMS are experienced differently by females of different backgrounds. For example, black females have the highest prevalence, duration, and severity of VMS (Avis et al., 2015; Gold et al., 2006; Lee et al., 2022; Lee & Keller‐Ross, 2025; Thurston et al., 2008). Further, females from lower socioeconomic statuses are more likely to experience VMS (Gold et al., 2006; Lee et al., 2022; Lee & Keller‐Ross, 2025). These important differences in social determinants of health highlight the need for further menopausal research that includes a diverse population.

There was a high degree of heterogeneity across included studies. Both LF and HF pooled estimates had substantial between‐study variability (*I* ^2^ = 97%), limiting the reliability of pooled effect sizes. This heterogeneity reflects differences in methodological design, including HRV measurement windows and analysis, inconsistent definitions, and reporting standards. Additionally, symptom assessment tools varied, with one study using unspecified structured questionnaires and self‐reports (Akiyoshi et al., 2011) and others employing specific instruments like the Menopause Rating Scale (Lee et al., 2011) and Hot Flash Weekly Weighted Symptom Score, in addition to self‐reports (Hoikkala et al., 2010). These inconsistencies made it challenging to directly compare or draw conclusions across studies and to generate consistent, reproducible insights into the relationship between autonomic function and VMS.

Finally, there are inherent limitations of HRV itself as a proxy for autonomic regulation. While HRV is widely used, it is an indirect measure that can be influenced by numerous physiological and contextual factors, such as respiratory patterns, circadian variation, emotional state, and posture (Laborde et al., 2017; Task Force of the European Society of Cardiology the North American Society of Pacing and Electrophysiology, 1996). Further, the HRV low frequency domain is not specific to one branch of the ANS and has been shown to have overlap on whether it is associated with sympathetic or parasympathetic tone (Eckberg, 1980, 1997; Rea & Eckberg, 1987). Additionally, variation in recording protocols, such as length of monitoring (e.g., 24‐h vs. 5‐min windows), time of day, and analysis techniques, can introduce further variability. These factors may affect both the interpretability and comparability of HRV data. Despite these limitations, HRV was chosen as the modality for this review because it remains a relatively noninvasive, accessible, and cost‐effective tool for capturing and interpreting autonomic control using simple ECG techniques.

### 4.3 Future directions

Moving forward, there is a critical need for both expanded evidence and methodological standardization. Future research should prioritize larger, independent, and longitudinal cohort studies to evaluate whether HRV changes are associated with VMS and whether these changes predict cardiovascular outcomes. Standardized HRV protocols are necessary and should include a minimum 5‐min supine period for an established baseline with beat‐to‐beat hemodynamics. Patients should also report their symptoms through the standardized Menopause Rating Scale for assessment of VMS. Additionally, symptom severity among women with VMS should be taken into consideration in future studies. Standardized HRV protocols will reduce heterogeneity, enhance cross‐study comparability, and ultimately clarify whether autonomic imbalance contributes to the link between VMS and cardiovascular health risk.

## 5 CONCLUSION

This systematic review explored whether VMS in menopausal and post‐menopausal women are associated with shifts in sympathetic and parasympathetic activity compared to menopausal and post‐menopausal women without VMS. Using HRV as a noninvasive biomarker, we observed that two out of the three eligible studies found differences in LF and LF/HF, which may reflect impaired baroreflex sensitivity and cardiovascular adaptability, but the pooled analysis was shy of statistical significance (*p* = 0.06). More studies with standardized HRV protocols, larger cohorts, and diversity in cohort characteristics are needed to better understand the relationship between autonomic function and VMS. HRV may represent a promising early marker of CVD risk in women with VMS, but further research is needed to clarify its clinical relevance.

## AUTHOR CONTRIBUTIONS

**Rashmin Hira:** Formal analysis; visualization. **Jaiden Uppal:** Data curation; formal analysis; visualization. **Paras Deol:** Conceptualization; data curation. **Danaka Porter:** Conceptualization; project administration. **Derek Exner:** Conceptualization; supervision. **Jacquie R. Baker:** Conceptualization; formal analysis; project administration; visualization. **Satish R. Raj:** Project administration; supervision.

## FUNDING INFORMATION

This work was supported in part by the National Center for Advancing Translational Sciences Award UL1 TR000445 (to Vanderbilt University).

## ETHICS STATEMENT

This study was exempt from Institutional Review Board approval.

## Supporting information


Figure S1.

Table S1.

## Data Availability

The data underlying this article will be shared on reasonable request to the corresponding author.
