# Supplementary material for: A systematic review of heart rate variability and menopausal vasomotor symptoms
Source: Physiol Rep. 2026 May 12;14(9):e70907. doi: 10.14814/phy2.70907 (PMC13167700; doi:10.14814/phy2.70907)
Supplement: Supplementary file 1 — Figure S1. [file PHY2-14-e70907-s002.docx]

**
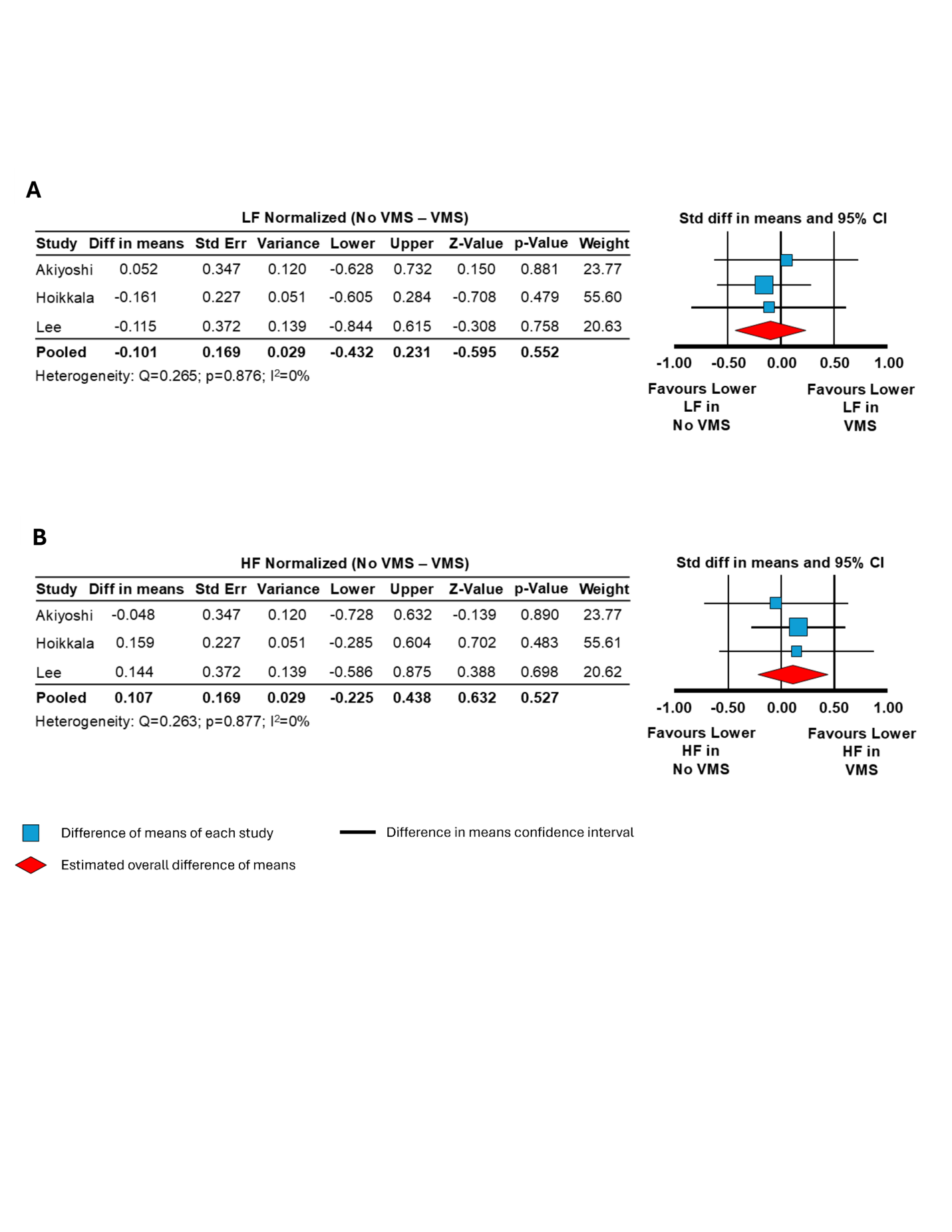
**

**Supplementary Figure 1 (S1).** Forest plots of difference in means for heart rate variability normalized frequency domain parameters in women with (N=187) and without (N=44) vasomotor symptoms (VMS). (A) Normalized low frequency power (nLF) in women with no VMS vs. with VMS (p=0.552). (B) Normalized high frequency power (nHF) in women with no VMS vs. with VMS (p=0.527).
